# An integrated framework for breast mass classification and diagnosis using stacked ensemble of residual neural networks

**DOI:** 10.1038/s41598-022-15632-6

**Published:** 2022-07-18

**Authors:** Asma Baccouche, Begonya Garcia-Zapirain, Adel S. Elmaghraby

**Affiliations:** 1 Department of Computer Science and Engineering, University of Louisville, Louisville, KY 40292 USA; 2 eVida Research Group, University of Deusto, 4800 Bilbao, Spain

**Keywords:** Breast cancer, Cancer imaging, Breast cancer, Cancer imaging, Biomedical engineering

## Abstract

A computer-aided diagnosis (CAD) system requires automated stages of tumor detection, segmentation, and classification that are integrated sequentially into one framework to assist the radiologists with a final diagnosis decision. In this paper, we introduce the final step of breast mass classification and diagnosis using a stacked ensemble of residual neural network (ResNet) models (i.e. ResNet50V2, ResNet101V2, and ResNet152V2). The work presents the task of classifying the detected and segmented breast masses into malignant or benign, and diagnosing the Breast Imaging Reporting and Data System (BI-RADS) assessment category with a score from 2 to 6 and the shape as oval, round, lobulated, or irregular. The proposed methodology was evaluated on two publicly available datasets, the Curated Breast Imaging Subset of Digital Database for Screening Mammography (CBIS-DDSM) and INbreast, and additionally on a private dataset. Comparative experiments were conducted on the individual models and an average ensemble of models with an XGBoost classifier. Qualitative and quantitative results show that the proposed model achieved better performance for (1) Pathology classification with an accuracy of 95.13%, 99.20%, and 95.88%; (2) BI-RADS category classification with an accuracy of 85.38%, 99%, and 96.08% respectively on CBIS-DDSM, INbreast, and the private dataset; and (3) shape classification with 90.02% on the CBIS-DDSM dataset. Our results demonstrate that our proposed integrated framework could benefit from all automated stages to outperform the latest deep learning methodologies.

## Introduction

Over years, breast cancer has remained the most frequently diagnosed non-skin cancer and the leading cause of death among females with a rate of 32% of total cancer cases^1^. According to the American Cancer Society, it is estimated that over 290,000 new cases will be reported and 43,780 women will die from breast cancer in 2022^2^. Early detection and diagnosis of breast cancer is the most effective way to treat this disease and reduce the mortality rate^3^.

Mammography has been proven the most reliable and preferred tool used by radiologists to screen and investigate suspicious breast lesions^4^. However, with the increase in the number of daily-screened mammograms, an efficient diagnostic methodology is necessary to assist doctors in the timely procedure of breast cancer. Thus, computer-aided diagnosis (CAD) systems, that perform computational image analysis, could provide a second suggestion and read to the final examination of the experts regarding the presence of breast cancer^5,6^.

A completely integrated CAD system would start its first stage, the detection and localization of suspicious lesions and distinguish between their types, i.e. mass, calcification, architectural distortion, etc. Then, at a second stage, the CAD system should perform a segmentation of the obtained region of interest (ROI) surrounding the breast lesion to recognize its anatomical contour and remove its tissue background without losing its shape precision. Finally, diagnostic information can be extracted regarding the lesion’s pathology to classify the decided lesion as either malignant or benign, and identify its characteristics such as tumor grading using Breast Imaging Reporting and Data System (BI-RADS) score, and shape categorization. As the automated procedure relies on connected stages, each output information must be generated precisely to generate a fast and accurate final decision. Therefore, different algorithms have been widely implemented in CAD systems, and the most commonly used are conventional machine learning classifiers and threshold-based methods that are based on handcrafted features^7–9^.

With the practical challenges that breast tumors offer due to their variation in size, shape, location, and texture, there has been a significant need to improve the overall performance of CAD systems and reduce false positive and negative cases. With the recent progress in computers and their enhanced computational capacity and speed, deep learning methodology has been broadly suggested in biomedical applications^10,11^ and particularly in CAD systems for mammography^12–15^.

In the last two decades, deep learning has shown growing success in many computer vision tasks and has proven a capability to overcome complex problems in the medical imaging domain. As a result, several works have been suggested and applied particularly in mammography, such as for tumors detection^16,17^, breast lesions segmentation^18,19^, and classification^20^.

Recently, deep learning models have exceeded the simple adaptation of convolutional neural networks (CNN) algorithms to present several advanced architectures that outperformed the image classification results^21,22^. The CNN architecture model was initially suggested for image classification and has been the base of many popular state-of-the-art architectures such as ResNet, AlexNet, EfficientNet, VGG, etc. Consequently, many works have studied and applied the recent classification models for breast lesions classification, and have been employed in CAD systems in different methodologies such as using ensemble learning^23,24^, transfer learning^25,26^, and fusion modeling^27–29^.

In this paper, we conduct the final stage in a CAD system, breast mass classification and diagnosis, using a stacked ensemble of neural network models. The proposed methodology completes our recent works conveyed for breast lesions detection and classification from entire mammograms, and is followed by a breast mass segmentation step. The work presents an integrated framework for the CAD system for breast cancer as the performance relies on three connected stages, and the current step generates the final decision about the breast mass’ pathology (i.e. benign or malignant), its BI-RADS category (i.e. score from 2 to 6), and its shape (i.e. oval, round, lobulated, or irregular).

The rest of the paper is organized as follows. First, the literature review of mass classification and diagnosis using deep learning, transfer learning and ensemble learning techniques is introduced in Sect. 2. In Sect. 3, details of our methodology are presented, including a description of the basic ResNet model and the suggested stacked ensemble of neural networks, followed by details about the used breast cancer datasets and preprocessing techniques. Then, in Sect. 4, we discuss the hyperparameters tuning applied for training the model, and present quantitative and qualitative results that are next compared with other works. We conclude the paper in Sect. 5 with a discussion of our proposed methodology and future works.

## Literature review

Several research studies have attempted to suggest machine learning methods for computer-aided diagnosis (CAD) systems to assist experts in their final diagnostic decisions and have focused on improving the results of breast mass classification in digital mammography. In this context, Dhahri et al.^30^ used a Tabu search to select the most significant features and then fed them into a K-Nearest Neighbors (KNN) algorithm to classify breast lesions into malignant or benign.

Since their development, many studies have given more attention to incorporating deep learning methods in CAD systems as they showed better efficiency than traditional CAD systems, which require extensive feature extraction. For instance, an end-to-end approach was developed by Shen et al.^31^ to classify digital mammograms into cancer or normal. The work presented a modern CNN structure using the VGG network and the residual network (ResNet), and achieved an area under the roc curve (AUC) of 0.91 on the CBIS-DDSM dataset and an AUC of 0.98 on the INbreast dataset. Another end-to-end model, called DiaGRAM, was built by Shams et al.^32^ that combined CNN and Generative Adversarial Networks (GAN). The work was conducted to classify mammograms as benign or cancerous and showed an accuracy of 89% on the DDSM dataset and 93.5% on the INbreast dataset. An improved deep learning method, called the DenseNet-II model, was invented in a work by Li et al.^33^ for the classification of benign and malignant mammograms. The model was applied to a private collection of mammograms and reached an accuracy of 94.55%. Accordingly, a model for mass classification was proposed by Zhang et al.^34^ that fused texton features with deep CNN features and achieved an accuracy of 94.30% on the CBIS-DDSM dataset. In another work by Muramatsu et al.^35^, a CNN model’s performance was improved by adding synthetic data generated from lung nodules in computed tomography (CT) using cycle GAN. The classification performance was tested on a DDSM dataset and achieved an accuracy of 81.4%. Recently, Chakravarthy et al.^36^ proposed a customized method that integrated deep learning with an extreme learning machine (ELM) for classifying abnormal ROI images into malignant or benign. The proposed work achieved a maximum accuracy of 97.19% on DDSM, 98.13% on the Mammographic Image Analysis Society (MIAS) dataset and 98.26% on INbreast datasets. In a recent work by Khan et al.^37^, a multi-view feature fusion (MVFF) based-CAD system was implemented to increase the performance of CNN by combining information from four views of mammograms in order to classify them into malignant or benign with an AUC of 0.84 on the CBIS-DDSM and mini-MIAS databases. A work by Jasti et al.^38^ tackled the problem of breast cancer diagnosis using first feature extraction by AlexNet model, feature selection by the relief algorithm, and simple machine learning models for disease categorization by KNN, random forest and Naïve Bayes.

Moreover, Kumar et al.^39^ suggested a classification framework for breast density using an ensemble of 4-class neural network classifiers. The work showed a classification accuracy of 90.8% on the DDSM dataset. A recent work by Yurttakal et al.^40^ has introduced a stacked ensemble of gradient boosting and deep learning models to classify breast tumors using DCE-MRI images. The work has shown an accuracy of 94.87% and an AUC value of 0.9728 on a private breast MRI dataset.

Besides ensemble learning methodology, transfer learning was also adapted with deep learning techniques to develop an approach for differentiation between benign and malignant breast cancer. Hence, in a work by Alkhaleefah et al.^41^, double-shot transfer learning (DSTL) was used by fine-tuning various pre-trained networks once on an ImageNet dataset, and another time on a larger dataset similar to the target dataset. The method was trained on the CBIS-DDSM and showed a better performance than single-shot transfer learning with an average AUC of 0.99 on the MIAS dataset and 0.94 on the BCDR dataset. Similarly, Falconí et al.^42^ used transfer learning on a NasNet Mobile model and fine tune on VGG models to classify mammogram images according to the BI-RADS scale achieving an accuracy of 90.9% on the INbreast dataset. Recently, a work by Medeiro et al.^43^ combined DenseNet201 and multi-perceptron layer (MLP) models to classify the pathology within BI-RADS levels 3 and 4 for malignancy of breast masses. The model achieved an accuracy of 63% surpassing the performance of a human expert by 9.0%. Another recent work by Tsai et al.^44^ proposed a deep neural network (DNN)-based model trained using block-based images segmented to classify BI-RADS categories for a private Asian dataset.

To accomplish an efficient mass classification and diagnosis procedure, researchers have shown that capturing texture and morphological characteristics could help doctors understand the nature of the breast tumor and assess its malignancy scale. For instance, research by Bi et al.^45^ showed that the probability of malignancy is highly correlated with the shape and morphology of a breast lesion. Therefore, several works have incorporated the segmentation stage to provide a complete, significant diagnosis. In a previous work by Tsochatzidis et al.^46^ modified convolutional layers of a CNN to integrate both input images and their corresponding segmentation maps in order to improve the diagnosis of breast cancer. The method was applied to DDSM-400 and CBIS-DDSM datasets and achieved a diagnosis performance of AUC of 0.89 and 0.86. Similarly, a dual convolutional neural network was suggested by Li et al.^47^, which computed the mass segmentation and simultaneously predicted the diagnosis results. The model contributed an improvement to the mass segmentation and cancer classification problem at the same time and achieved an AUC of 0.85 on the DDSM dataset and 0.93 and the INbreast dataset.

Recently, most of the developed CAD systems have automated the breast cancer diagnosis procedure that gets an entire mammogram image and returns the final diagnosis. Thus, many studies have integrated the first stage of identifying the suspicious region of breast lesions and based on its automated output, performed the segmentation and classification tasks. For instance, Sarkar et al.^48^ proposed an automated CAD system that detects suspicious regions of potential lesions using a deep hierarchical prediction network and then classifies them into mass or non-mass, and finally into malignant or benign using a CNN structure. The work was tested and achieved an accuracy of 98.05% on the DDSM dataset and 98.14% on the INbreast dataset. Another fully automated system by Dhungel et al.^49^ for breast mass classification integrated mass detection and segmentation in a complete CAD system. The methodology used a multi-scale deep belief network (m-DBN) classifier followed by a cascade of CNNs and random forest classifiers for false positive reduction for mass detection, a conditional random field (CRF) for mass segmentation, and a multi-view deep residual neural network (mResNet) for mass classification. The proposed work achieved an AUC of 0.8 on the INbreast dataset. Another recent work by Singh et al.^50^ presented an automatic workflow that detects breast tumor regions from mammograms using the Single Shot Detector (SSD), and then outlines its segmented mask using conditional Generative Adversarial Network (cGAN) that was finally used for shape classification using a CNN. The framework achieved an overall accuracy of 80% for the shape classification. Similarly, Al-Antari et al.^51^ proposed a fully integrated CAD system for digital mammograms via deep learning techniques. It started with a mass detection using the You-Only Look Once (YOLO) architecture model, then performed a mass segmentation on the detected regions using a Full resolution convolutional network (FrCN), and finally classified the detected and segmented masses into benign or malignant using a CNN model. The entire framework had an overall classification accuracy of 95.64% on the INbreast dataset. The mass classification step was differently solved in recent work by Al-Antari et al.^52^ that separately adopted three conventional deep learning models including regular feedforward CNN, ResNet-50, and InceptionResNet-V2. The work achieved a maximum accuracy of 95.32% on the INbreast dataset.

Inspired by the continuous success of the CNN model and its variations for breast mass classification, we propose a stacked ensemble of residual network (ResNet) models to classify and diagnose previously detected and segmented mass lesions. The proposed model uses three different architectures of the ResNet model, ResNet50V2, ResNet101V2, and ResNet152V2 that are transferred and fine-tuned on our mammography datasets. The models’ layers are stacked together and reconfigured into an entire model for an overall classification and diagnosis of 1) the pathology as malignant or benign; the BI-RADS category as assessment score from 2 to 6; and 3) the lesions’ shape as round, oval, lobulated, or irregular.

The contributions of this paper are as follows:

- We demonstrate the efficiency of a type of ensemble modeling technique—a Stacked ensemble of neural networks—in enhancing the individual performance of one of the SOTA models for mammography image classification
- We show that an integrated framework of CAD system for breast cancer, where detection and segmentation results are highlighted, is essential for a precise classification and diagnosis
- We present a complete breast cancer diagnosis with malignancy classification, BI-RADS assessment score and tumor shape categorization

The presented work will serve as the last stage of an integrated framework for a breast cancer CAD system. The previous stages were proposed in recent works by Baccouche et al. where the detection and classification step was first applied using a YOLO-based fusion model to localize and identify suspicious breast lesions as mass or classification^28^, and then using only the detected masses, a Connected-UNets model was suggested for breast mass segmentation improved with combining real and synthetic data generated by CycleGAN model^53^.

The paper is inspired by ensemble model learning and fusion modeling that showed high efficiency in many recent studies. The suggested methodology was performed on two most popular public mammography datasets: Curated Breast Imaging Subset of Digital Database for Screening Mammography (CBIS-DDSM) and INbreast, and on a private collection of mammograms.

## Material and methods

In this study, we propose a stacked ensemble of models to classify and diagnose detected and segmented breast masses. The base model comes from the ResNet architecture and its variations. Our methodology employs different strategies: transfer learning, stacked ensemble learning, and image data augmentation.

### ResNet base model: transfer learning and fine-tuning

Since its introduction, ResNet is a deep CNN architecture suggested by He et al.^54^ that have been one of the recent architectures that has known common success in medical imaging applications^55,56^. ResNet uses residual blocks with skip connections between layers to bypass a few convolution layers at a time. This architecture accelerated the convergence of a larger number of deep layers, and consequently it has been found efficient to provide a compact representation of input images and improve the classification task performance^27^. The ResNet has some common architectures such as ResNet-50, 101, and 152,^46^ which indicate the number of deep layers. Alternatively, ResNet architecture presented an improved version of ResNetV2 by He et al.^57^, where the last ReLU was removed to clear the shortcut path using a simple identity connection as shown in Supplementary Fig. 1.

Our methodology employs three pre-trained ResNetV2 architectures, detailed below in Supplementary Table 1. Training a deep learning model often requires a large amount of annotated data that helps optimize the high number of parameters and computations needed in the architecture. However, the limited size of medical imaging datasets is usually available that suffer from either missing labels or imbalanced data distribution. To overcome these challenges, transfer learning has been a common solution used in many recent medical image applications^58,59^ by training a model on a large and diverse dataset (i.e. ImageNet, MSCOCO, etc.) to capture universal features like curves, edges, and boundaries in its early layers that are relevant for image classification. After that, the pre-trained model should be alerted and fine-tuned on a custom and specific dataset to reflect the final classification. This procedure provides a fast and generalizable training of small datasets and avoids the overfitting problem that deep learning commonly suffers from.

**Figure 1:**
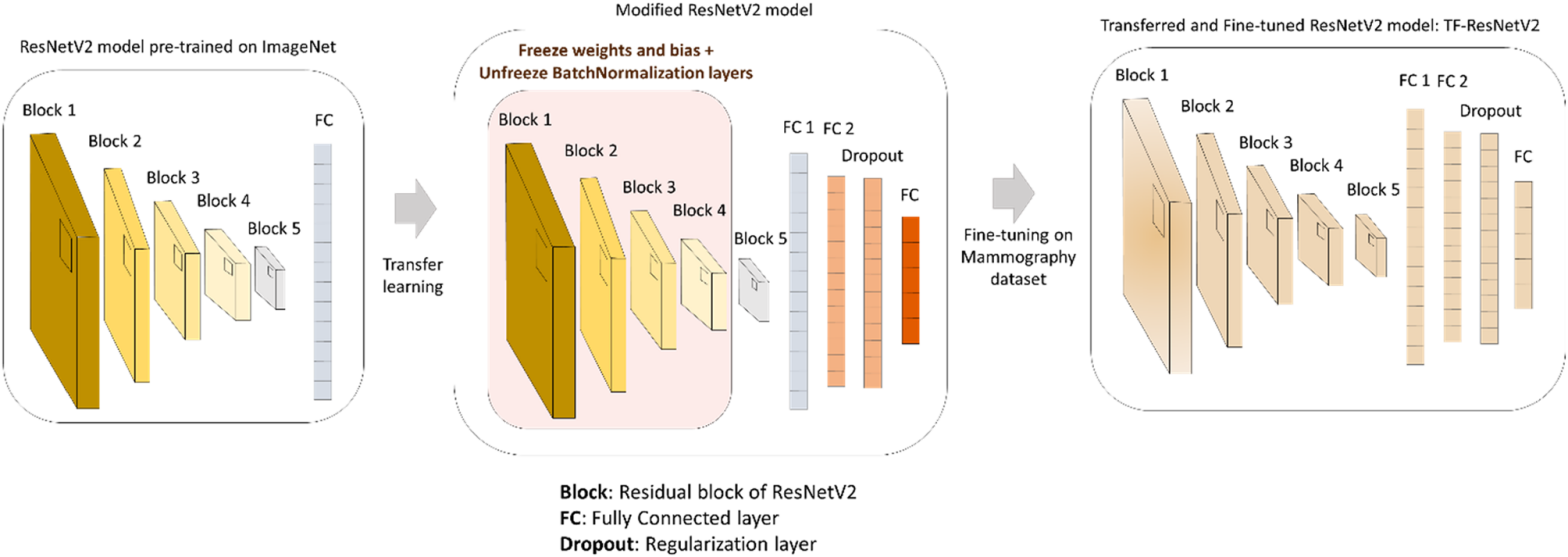
Framework of the classification base model: a TF-ResNetV2 model which is a ResNetV2 model pre-trained on ImageNet data and modified and fine-tuned on mammography dataset.

As Fig. 1 indicates, we apply transfer learning to the base architecture ResNetV2 for our proposed methodology to become a TF-ResNetV2. The model was initially pre-trained on ImageNet, and then the first four residual blocks of layers were frozen except for the BN layers that needed to be retrained in order to improve the training convergence. After that, the entire architecture was modified by adding another FC layer with a size of 1024, followed by a dropout regularization layer to maintain a generalization aspect for the training. A new final FC layer was placed according to the number of classes for each classification task and the entire TF-ResNetV2 is re-trained.

### Stacked ensemble of ResNet models for breast mass classification

Ensemble learning has been considered efficient to improve the classification task results. Combining weaker classifiers to create a better final classification prediction has been adopted by either bagging, boosting, or stacking models. While bagging is achieved by learning independently from different models and then averaging the predictions, boosting happens by sequentially learning from homogenous learners and iteratively combining them into a final model. On the other hand, stacking has been considered a way to learn different weak learners in parallel and combine them into a meta-model that is later trained to achieve the classification prediction^60^.

**Figure 2:**
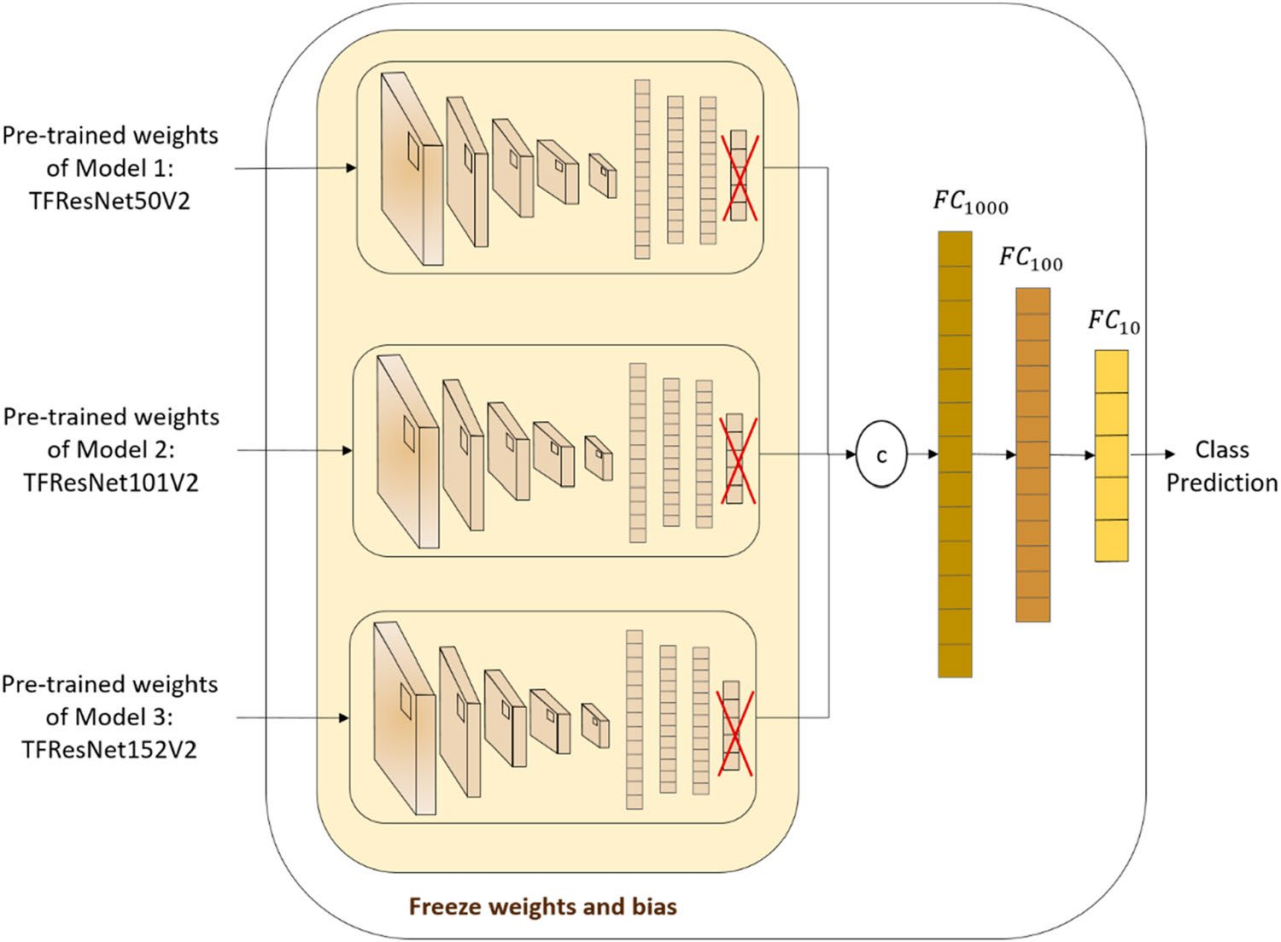
Framework of the classification Stacked Ensemble of ResNet models.

We propose a stacked ensemble of three different ResNet models to conduct our classification tasks. After removing the last FC layer of each ResNetV2 architecture, a two-layers network is considered as a meta-classifier model that concatenates the three models’ layers, and stacks three different FC layers of sizes 1000, 100 and 10, coupled with activation functions Sigmoid and ReLU. As shown in Fig. 2, after training independently ResNet50V2, ResNet101V2 and ResNet152V2, pre-trained weights of each model were extracted as images features of size 1024 based on previous layer predictions and considered as new input of the entire stacked ensemble of ResNet models for the final class prediction.

### Integrated framework: mass detection, segmentation and classification

**Figure 3:**
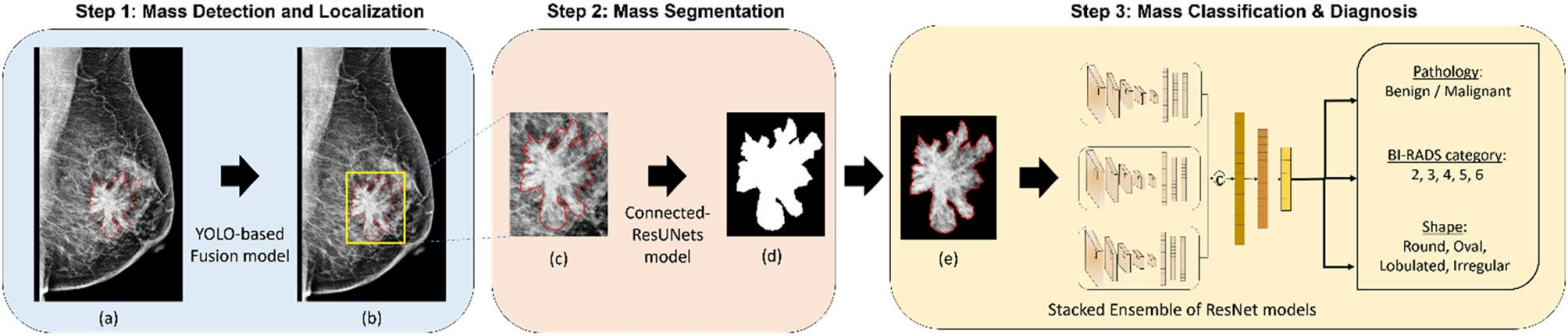
The proposed integrated CAD framework. (**a**) Original mammogram with ground truth of mass (red), (**b**) Detected ROI of mass (yellow) superimposed on the original mammogram, (**c**) Detected ROI mass obtained with ground truth (red), (**d**) Output segmented binary mask of ROI mass, and (**e**) Segmented ROI mass with marked tissue.

Our final framework should now be complete with all automated steps for breast cancer analysis and diagnosis. Therefore, as shown in Fig. 3, the integrated framework detects and localizes breast masses in a first step using YOLO-based fusion models^28^, which only require an entire mammogram image and outputs bounding boxes around specious lesions. The model was evaluated and provided a maximum detection accuracy of 98.1% for mass lesions. The next step should segment the detected ROI of breast masses and generate a binary mask image where only the boundary of the lesions is visible. The second step is achieved using the proposed Connected-UNets model^53^ that was improved by synthetic data, which is generated by CycleGAN. The segmentation step was conducted on ROI images scaled to optimal size of 256 × 256 pixels.

The evaluation showed a high Dice score of 95.88% and Intersection over Union (IoU) of 92.27%. After that, the segmented and detected ROI of breast masses generated with masked tissue is used for the third and final classification step. The stacked ensemble of ResNet models is trained independently on the input ROI masses for each classification task to finally predict the pathology as either malignant or benign, the BI-RADS category with an assessment score between 2 to 6, and the shape as either round, oval, lobulated or irregular.

### Datasets

Similar to our previous works, we evaluate the proposed classification methodology on two public datasets, the CBIS-DDSM and INbreast datasets, and an independent private dataset. The CBIS-DDSM dataset^61^ is an updated and standardized version of the Digital Database for Screening Mammography (DDSM), where images were reviewed by radiologists to eliminate inaccurate cases and converted from the Lossless Joint Photographic Experts Group (LJPEG). It contains 2907 mammograms from 1555 unique patients, where 1467 are mammograms with mass lesions acquired with two different views (i.e. MLO and CC). Original mammograms have an average size of 3000 × 4800 pixels and are associated with their pixel-level annotation and class labels (i.e. Pathology, BI-RADS category and Shape).

The INbreast dataset^62^ is a public database of Full-Field Digital Mammography (FFDM) images in DICOM format. It contains 410 mammograms from 115 unique patients where only 107 cases present mass lesions in both MLO and CC views. Original mammograms have an average size of 3328 × 4084 pixels and include pixel-level annotation and class labels (i.e. Pathology and BI-RADS category).

**Figure 4:**
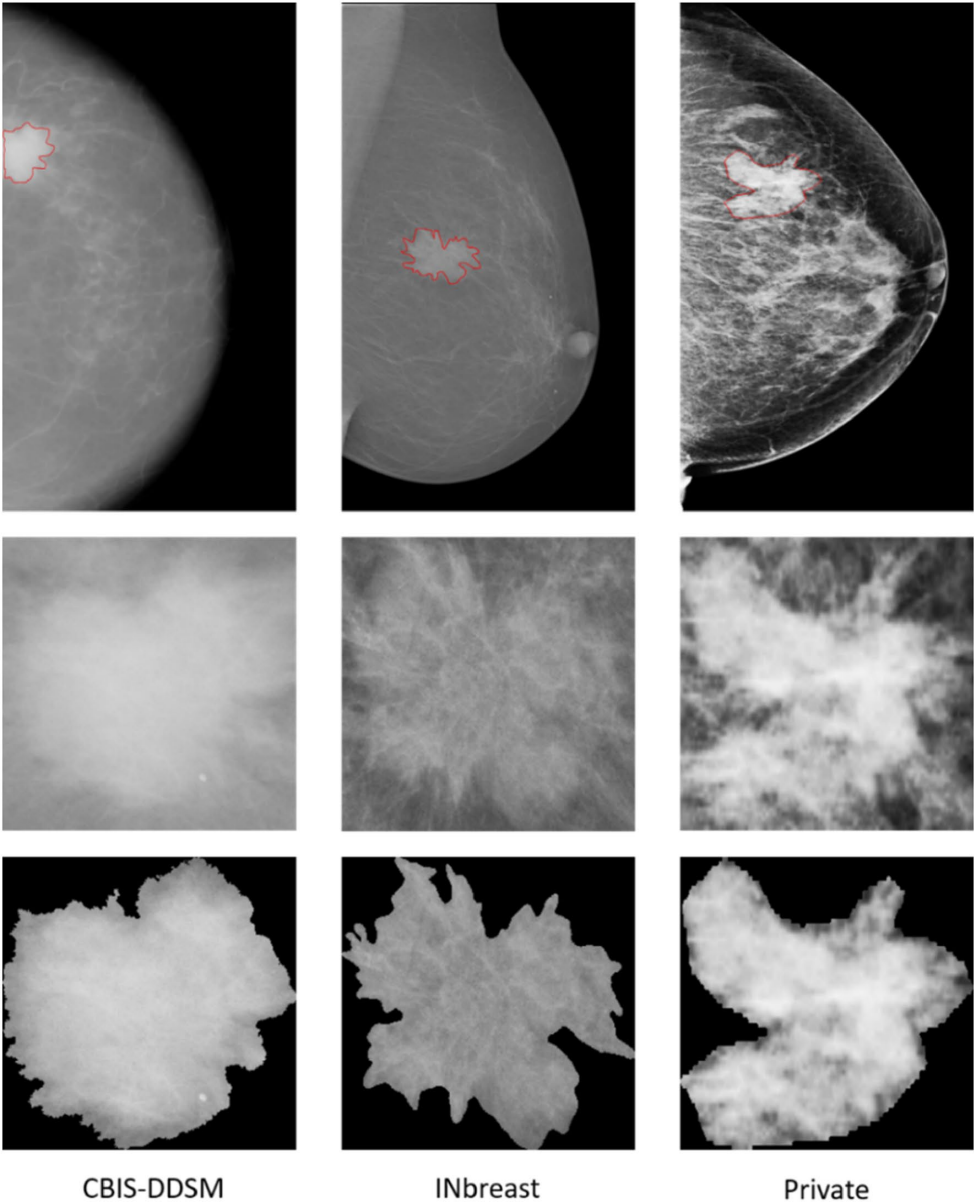
Samples of entire mammograms and ROI of a mass detected and segmented from different mammography datasets with ground-truth of location and contour of mass in red.

The private dataset is a collection of mammograms from the National Institute of Cancerology (INCAN) in Mexico City and contains stages 3 and 4 of breast cancer with 389 cases from 208 unique patients having mass lesions. Images have an average of 300 × 700 pixels acquired from different views (i.e. CC, MLO, ML and AT), and include associated pixel-level annotation and class labels (i.e. Pathology and BI-RADS category). Figure 4 illustrates samples of original mammograms and their ROI masses compared to the detected and segmented ROI masses from different datasets.

As the datasets were explored continuously during the previous studies, the original mammograms that included mass and calcification cases were used during the first step of detection and localization; therefore detected ROIs of only mass cases were retained for the second step of segmentation. It is fair to mention that some mammograms have multiple ROIs and hence the number of detected and segmented ROI masses used for the third step of classification and diagnosis may vary. Due to the limited amount of ROI masses in each dataset, raw ROIs data was augmented four times by rotating them with the angles Δθ = {0°, 90°, 180°, 270°}, and transformed twice differently using the Contrast Limited Adaptive Histogram Equalization (CLAHE) method. Table 1 details the data distribution of each mammography dataset regardless of the class labels.

**Table 1:** General datasets distribution.

Dataset	Raw MGs Data	Raw ROIs Data	Augmented Data (ROIs*6)
CBIS-DDSM	1467	1467	8802
INbreast	107	112	672
Private	389	638	3828

Moreover, each mammography dataset has a different quality of images in terms of pixel quality, existing annotated labels and class distribution, as detailed in Tables 2, 3 and 4. Only the CBIS-DDSM dataset includes true class labels for lesions’ shape. Accordingly, the INbreast dataset indicates cases with a BI-RADS score from 2 to 6, however, the CBIS-DDSM dataset presents cases in BI-RADS category 2 to 5, and the private dataset has only malignant cases as it acquired breast cancer cases from only stages 3 and 4. Consequently, all mammograms from the private dataset fall into BI-RADS categories 4 and 5.

**Table 2:** Pathology class labels distribution.

Dataset	Pathology	
	benign	Malignant
CBIS-DDSM*	4500	4302
INbreast**	150	522
Private	0	3830

*Cases with Benign_without_callback are considered Benign.

**Cases with BI-RADS score > 3, are considered Malignant otherwise Benign.

**Table 3:** Shape class labels distribution.

Dataset	Shape			
	Round	Oval	Lobulated	Irregular
CBIS-DDSM	804	2040	2112	3846
INbreast	NA	NA	NA	NA
Private	NA	NA	NA	NA

**Table 4:** BI-RADS category class labels distribution.

Dataset	BI-RADS				
	Category 2	Category 3	Category 4	Category 5	Category 6
CBIS-DDSM	792	1938	2328	3402	0
INbreast	144	78	126	276	48
Private	0	0	1627	2195	0

## Results

All experiments for the proposed methodology were conducted using Python 3.6 on a PC with the following specifications: Intel(R) Core (TM) i7-8700K processor with 32 GB RAM, 3.70 GHz frequency, and one NVIDIA GeForce GTX 1090 Ti GPU.

### Data preparation

**Figure 5:**
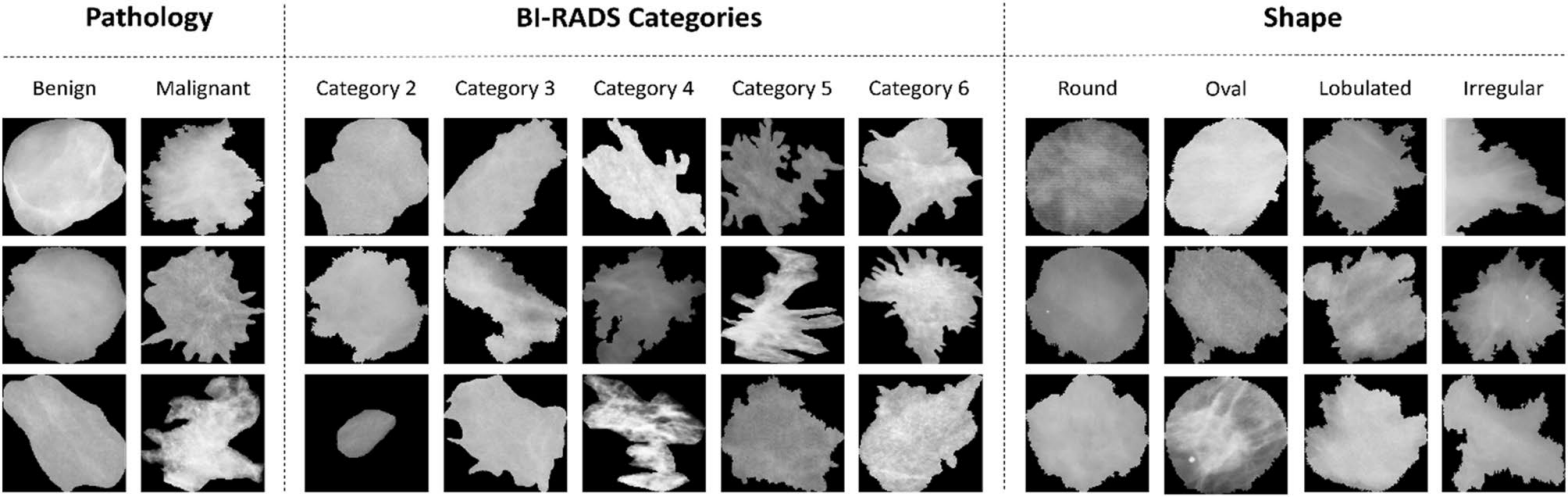
Samples of detected and segmented ROI masses for each class within different classification tasks from different mammography datasets.

Mammograms are often collected using a scanning machine of digital X-ray mammography that usually compresses the breast and consequently, it degrades the images. Therefore, we apply preprocessing techniques to remove the additional noise and correct the data using a histogram equalization that smooths the pixel distribution. Furthermore, the pre-trained ResNet models require an input image size of 224 × 224; therefore, we resize the detected and segmented ROIs from 256 × 256 using an inter-area resampling interpolation. Finally, all images are normalized to a range of [0, 1]. Samples of input data for each classification class are illustrated in Fig. 5 where ROIs are distributed according to different class labels from the mammography datasets.

### Evaluation metrics

All classification tasks are evaluated overall using the accuracy, and area under the curve (AUC) that reflect the performance of the model while considering the unbalanced mammography datasets. Particularly, for pathology classification, which presents a binary-classes case, we use three additional metrics called sensitivity, specificity scores, and F1-score, as shown in Eqs. 1, 2, and 3. The F1-score is a coefficient that represents a harmonic average between the specificity and sensitivity, where its maximum score of 1 indicates perfect specificity and sensitivity and of 0 the worst performance. Moreover, the accuracy score is a rate of correct predictions over all cases as detailed in Eq. (4) where TP, TN, FP, and FN are defined per predicted class to represent the number of true positive, true negative, and false positive, and false negative predictions, respectively.

1 $$Sensitivity=\frac{TP}{TP+FN}$$

2 $$Specificity=\frac{TN}{TN+FP}$$

3 $$F1-score=\frac{2\times TP}{2\times TP+FP+FN}$$

4 $$Accuracy=\frac{True prediction}{Total cases}=\frac{TP+TN}{TP+FP+TN+FN}$$

In pathology classification, positive refers to the malignant class and negative refers to the benign class. In BI-RADS category and shape classification, macro averaging is used to compute the accuracy and the AUC scores. Consequently, a confusion matrix can be driven from these measurements to show the tradeoff between the true and predicted class labels.

### Hyperparameters tuning

Extensive experiments with different variations in hyperparameters were conducted to select the best parameters for the base ResNetV2 model. Considering their effect on the classification performance, only hyperparameters detailed in Table 5 were tuned to select the best-configured network that outperforms the evaluated networks on all mammography datasets.

**Table 5:** Hyperparameters for the ResNetV2 base model.

Hyperparameters	Values explored	Description
Batch size	32, 64	mini-batch training size
Epochs	20, 30, 50	Number of training epochs
Dropout	0%, 20%, 30%	% of neurons of hidden layers “dropped” for regularization
LR	10^–1^, 10^–2^, 10^–3^	Learning rate for the Adam optimizer
Smoothing	0% 20%, 25%	% of label smoothing for the loss function

For all datasets, we randomly split images for each class into groups of 80% for training, and 20% divided equally between testing and validation sets. In each experiment, the same trainable parameters were used and each hyperparameter was varied accordingly. For all datasets and classification tasks, we used Adam optimized and evaluation was reported with a weighted accuracy score to reflect the class imbalance during the training and testing. The loss function was employed according to the classification task, a Binary Cross-entropy function for binary classes and a Categorical Cross-entropy for multiple classes. In both cases, a label smoothing technique for regularization to help overcome overfitting and provide a generalized model. The technique works by explicitly updating the labels during the loss function and decreasing the model’s confidence when it starts diverging^63^. In addition, training was monitored using a method that reduces the learning rate if the accuracy stops improving. Thus, we applied the stated strategy with a factor of 0.5 when the accuracy did not improve after two iterations. Conclusively, the best evaluation was reported with a batch size of 32, 30 epochs, a dropout rate of 30%, a learning rate of 10^–2^, and a smoothing label of 25%.

### Quantitative classification results

The proposed breast mass classification model was trained and compared to single base models for each presented task on the different mammography datasets. We also compared the stacked ensemble of models to a conventional average of different models’ weights with an XGBoost classifier.

#### Pathology classification

As shown in Tables 6, 7, and 8, the pathology classification results are compared between different models respectively for CBIS-DDSM, INbreast, and private datasets. It is reasonable to mention that because the private dataset includes only malignant cases, we trained and tested the model on a combination of all datasets.

**Table 6:** Pathology classification results on the CBIS-DDSM dataset.

Model	Accuracy	Sensitivity	Specificity	F1-score	AUC
Model1: ResNet50V2	89.97	0.89	0.91	0.9	0.9
Model2: ResNet101V2	93.57	0.92	0.95	0.94	0.93
Model3: ResNet152V2	92.11	0.92	0.92	0.92	0.92
Average Weights of Model1, Model2 and Model3 + XGBoost Classifier	91.04	0.85	0.98	0.91	0.91
Stacked Ensemble of models	95.13	0.93	0.97	0.95	0.95

**Table 7:** Pathology classification results on the INbreast dataset.

Model	Accuracy	Sensitivity	Specificity	F1-score	AUC
Model1: ResNet50V2	98.52	1.0	0.93	0.97	0.96
Model2: ResNet101V2	95.58	1.0	0.80	0.93	0.9
Model3: ResNet152V2	96.6	1.0	0.90	0.91	0.94
Average Weights of Model1, Model2 and Model3 + XGBoost Classifier	97.9	1.0	0.96	0.98	0.97
Stacked Ensemble of models	99.2	1.0	0.98	0.99	0.99

**Table 8:** Pathology classification results on the Private dataset.

Model	Accuracy	Sensitivity	Specificity	F1-score	AUC
Model1: ResNet50V2	92.83	0.94	0.90	0.91	0.92
Model2: ResNet101V2	94.18	0.94	0.95	0.93	0.94
Model3: ResNet152V2	94.60	0.94	0.94	0.94	0.94
Average Weights of Model1, Model2 and Model3 + XGBoost Classifier	94.89	0.94	0.97	0.93	0.94
Stacked Ensemble of models	95.88	0.93	0.97	0.95	0.96

**Figure 6:**
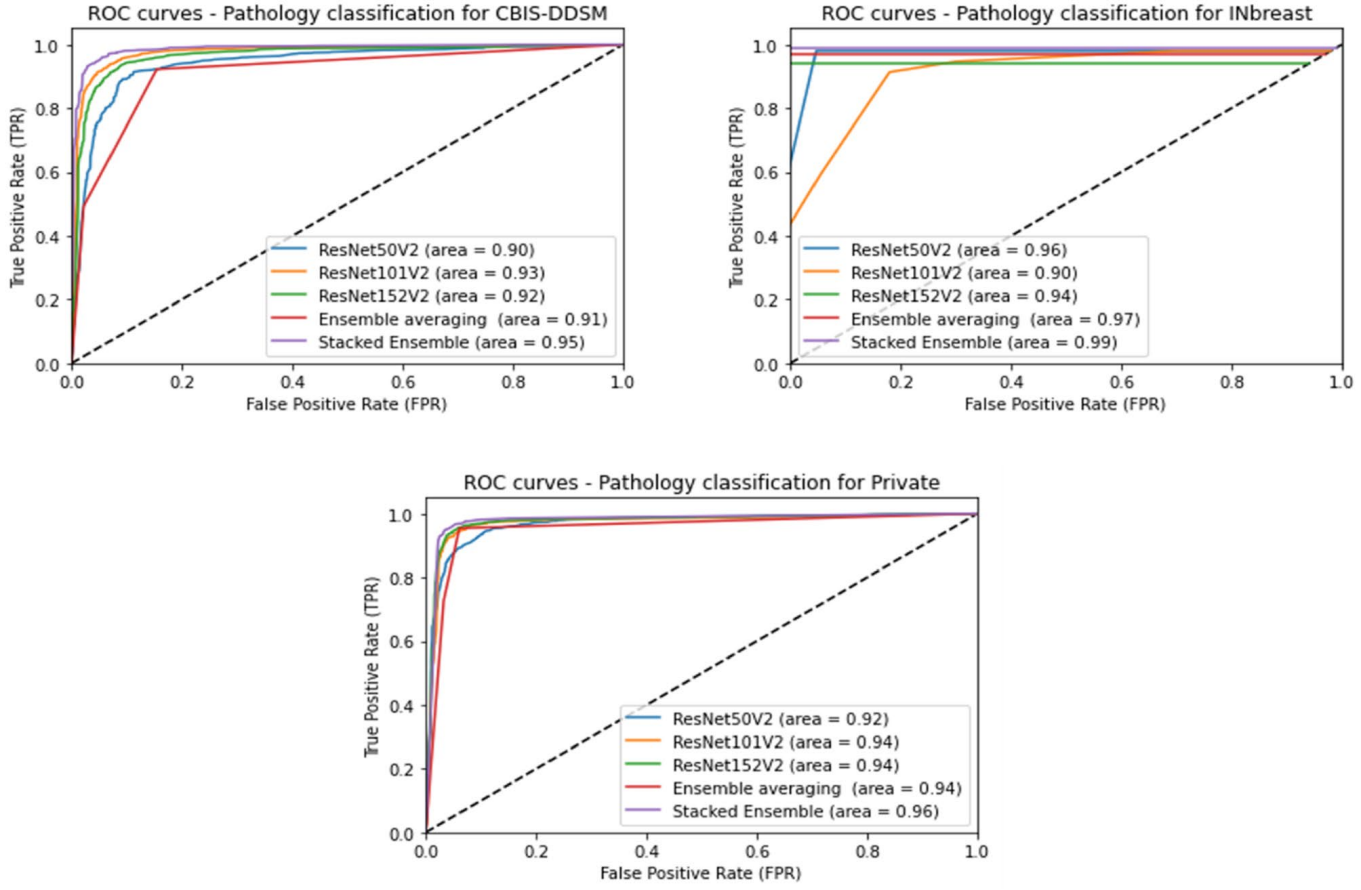
Performance of pathology classification using different models in terms of ROC curves and AUC score.

The comparative results show that the proposed stacked ensemble of models performs better than the base ResNet models having different numbers of deep layers (i.e. ResNet50V2, ResNet101V2 and ResNet152V2). Accordingly, our proposed methodology outperformed the average ensemble of models with an XGBoost classifier that performed slightly better than individual models. We notice a high accuracy of 95.13% on the CBIS-DDSM dataset, 99.2% on the INbreast dataset, and 95.88% on the private dataset. Besides, our proposed model achieved a high sensitivity rate of 0.93 on the CBIS-DDSM dataset, 1.0 on the INbreast dataset, and 0.93 on the private dataset. Consequently, the results emphasize generally the advantage of the ensemble learning technique in improving the classification performance, and particularly the improvement achieved by the stacking method using deep learning models. Moreover, the pathology classification performance was compared against the different models using the AUC over the test sets of all datasets. Figure 6 shows plots of the Receiver Operating Characteristic (ROC) curves of the True positive Rate (TPR) against the False Positive Rate (FPR), and we notice that the proposed model outperformed all experimental techniques with an AUC of 0.95 for the CBIS-DDSM dataset, 0.99 for the INbreast dataset, and 0.96 for the private dataset.

#### BI-RADS category classification

Results that are shown in Tables 9, 10 and 11 for BI-RADS category classification illustrate the comparison between different models for all mammography datasets. As mentioned in the section on Datasets description, each dataset has different class labels that vary from category 2 to category 6.

**Table 9:** BI-RADS category classification results on the CBIS-DDSM dataset.

Model	Accuracy	AUC
Model1: ResNet50V2	80.07	0.93
Model2: ResNet101V2	77.12	0.92
Model3: ResNet152V2	80.08	0.93
Average Weights of Model1, Model2 and Model3 + XGBoost Classifier	79.48	0.85
Stacked Ensemble of models	83.84	0.94

**Table 10:** BI-RADS category classification results on the INbreast dataset.

Model	Accuracy	AUC
Model1: ResNet50V2	98.0	0.94
Model2: ResNet101V2	98.0	0.97
Model3: ResNet152V2	96.1	0.92
Average Weights of Model1, Model2 and Model3 + XGBoost Classifier	97.1	0.99
Stacked Ensemble of models	99.0	1.00

**Table 11:** BI-RADS category classification results on the Private dataset.

Model	Accuracy	AUC
Model1: ResNet50V2	91.91	0.91
Model2: ResNet101V2	92.43	0.92
Model3: ResNet152V2	94.25	0.94
Average Weights of Model1, Model2 and Model3 + XGBoost Classifier	92.95	0.93
Stacked Ensemble of models	96.08	0.95

**Figure 7:**
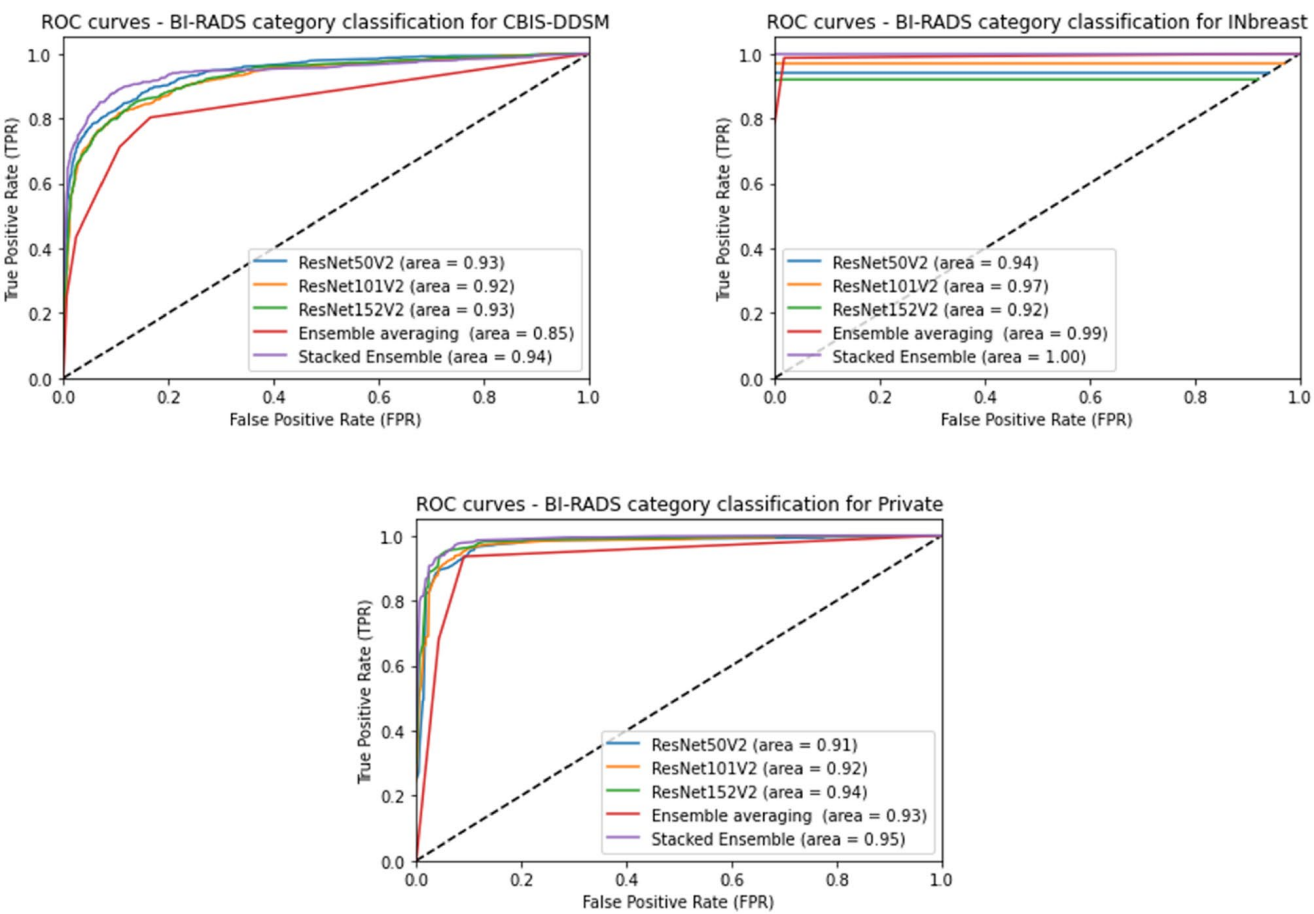
Performance of BI-RADS classification using different models in terms of ROC curves and AUC score.

The classification results presented above demonstrate a clear improvement of the performance using our proposed stacked ensemble of models compared to the basic models with an accuracy of at least 3.78% on the CBIS-DDSM dataset, and 1% on the INbreast dataset, and 1.83% on the private dataset. Moreover, our methodology achieved a better AUC score than the average ensemble model with an XGBoost classifier where we notice a high AUC of 0.94 for the CBIS-DDSM dataset, 1.00 on the INbreast dataset, and 0.95% on the private dataset. This can be confirmed with a visual comparison of ROC curve plots between employed models as illustrated in Fig. 7.

#### Shape classification

Lastly, the proposed model was trained on the CBIS-DDSM dataset for classifying the shape of breast masses, as it is the only dataset that possesses shape annotation by experts. Equivalently, all trained models were tested, and a comparison is shown in Table 12. Undoubtedly, our suggested stacked ensemble of models had the highest accuracy score of 90.02% among the employed models, which improved the performance of separate models notably with 1.7% and remarkably with 10.66% compared to the average ensemble of models with an XGBoost classifier.

**Table 12:** Shape classification results on the CBIS-DDSM dataset.

Model	Accuracy	AUC
Model1: ResNet50V2	75.51	0.90
Model2: ResNet101V2	89.90	0.95
Model3: ResNet152V2	88.32	0.97
Average Weights of Model1, Model2 and Model3 + XGBoost Classifier	79.36	0.84
Stacked Ensemble of models	90.02	0.98

**Figure 8:**
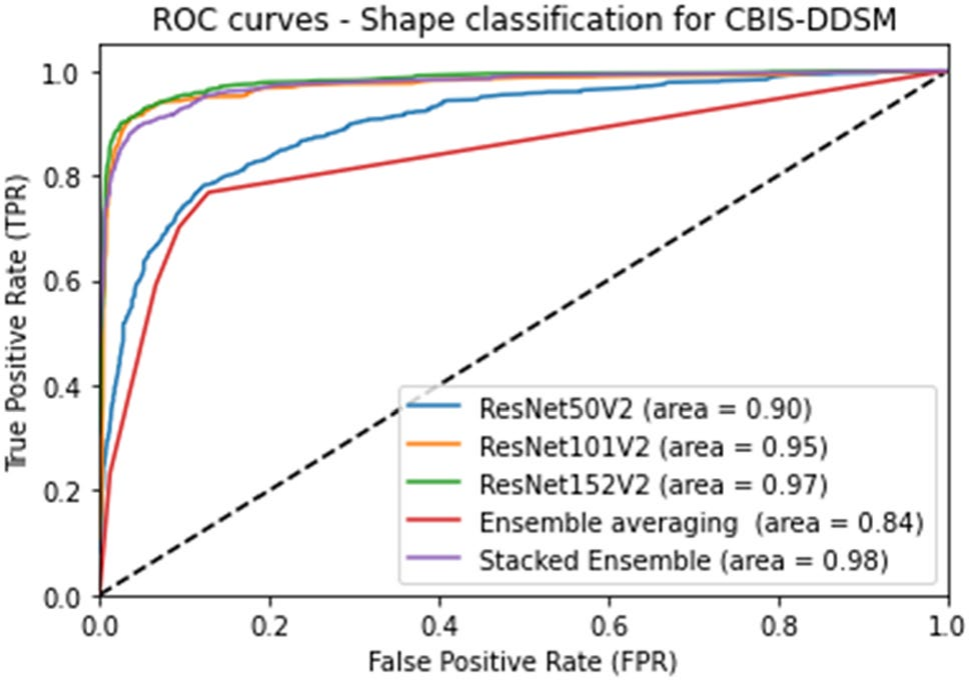
Performance of Shape classification using different models in terms of ROC curves and AUC score.

Furthermore, Fig. 8 presents a comparison of ROC curve plots for the different employed models and represents the AUC score accordingly. We notice that our proposed model had the highest AUC of 0.98 among the presented models, which was close to the ResNet152V2 performance but with a slightly better accuracy rate.

### Qualitative classification results

Previous comparison results highlighted that our proposed methodology yielded the best results for the different classification tasks. Consequently, we analyzed the classification prediction between different datasets using the confusion matrix that summarizes the results across the class labels.

**Figure 9:**
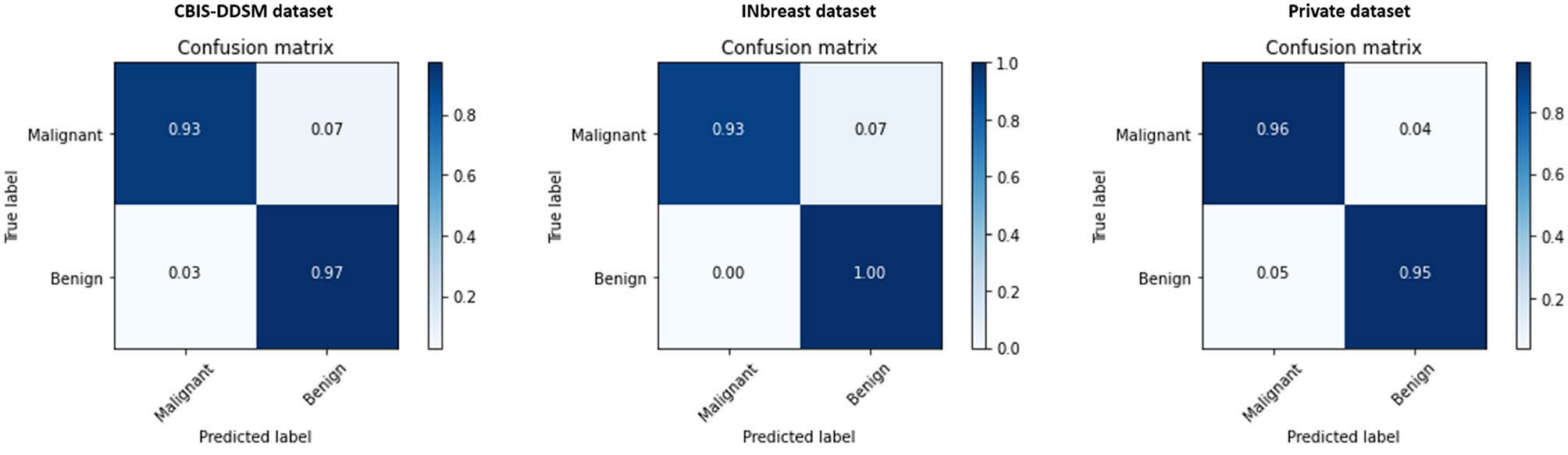
Confusion matrix of the stacked ensemble of models for the pathology classification on the mammography datasets.

**Figure 10:**
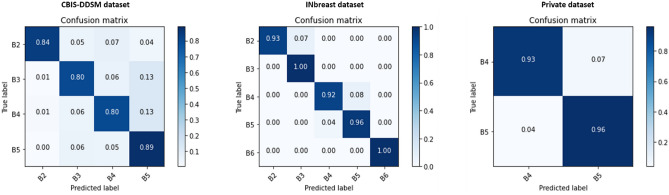
Confusion matrix of the stacked ensemble of models for the BI-RADS category classification on the mammography datasets.

**Figure 11:**
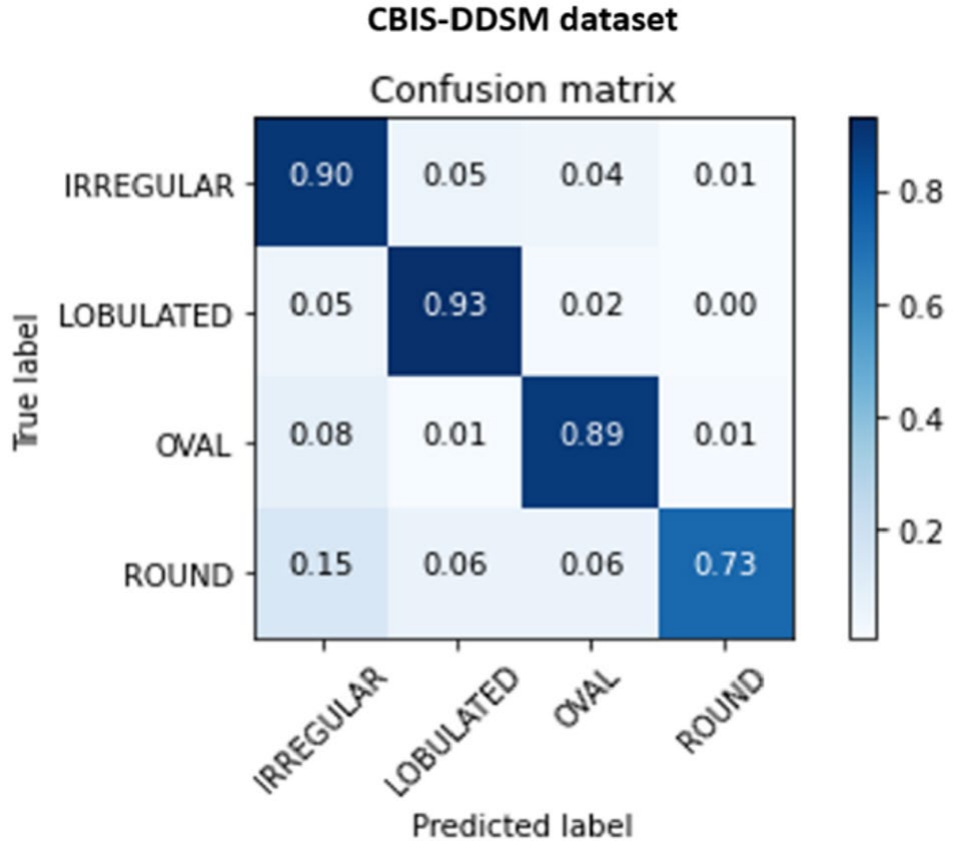
Confusion matrix of the stacked ensemble of models for the Shape classification on the mammography datasets.

Figures 9, 10 and 11 respectively present the normalized confusion matrix plots for each classification problem. As indicated below, the INbreast dataset had the best pathology classification tradeoff between malignant and benign classes, and this can be explained by the high-quality resolution of the mammograms collected in FFDM format that helps distinguish between the two class labels. The private dataset had also a remarkable confusion matrix with close recall and precision scores, which were similar to the CBIS-DDSM dataset’s performance.

Moreover, the INbreast dataset had the best BI-RADS categorization tradeoff with a notable prediction per class from 0.92 to 1.0. Concerning the private dataset, it has only two BI-RADS categories 4 and 5, and we notice a similar satisfying confusion matrix with prediction scores of 0.93 and 0.96. The CBIS-DDSM dataset has a slightly worse tradeoff for the BI-RADS category classification and this is due to the low resolution of deteriorated ROI images from the digitized X-rays mammograms. the confusion matrix shows values from 0.80 to 0.89 and we notice that the four categories have a close prediction score due to the similarity of the pixel distribution caused by the quality presented in the public dataset.

Finally, the confusion matrix for the shape classification showed an overall sufficient tradeoff between the class labels. We observe similar predicted results for the irregular and lobulated cases with a maximum value of 0.93, and this can be interpreted by the close appearance of the two lesions’ shapes. However, oval and round cases had worse results, and in particular, the round class label had a performance score of 0.73.

Additionally, we reported the classification results of the final integrated CAD system using the segmentation step and compared it to the results of the proposed method without using the segmented ROIs. Table 13 shows a better classification performance for each task on all mammography datasets, where the pathology classification demonstrated an improvement of 4.26% on the CBIS-DDSM dataset, 4% on the INbreast dataset, and 5.5% on the private dataset. Accordingly, the BI-RADS category classification presented enhanced performance using the detected and segmented images with an accuracy difference of 3.69% on the CBIS-DDSM dataset, 1.5% on the INbreast dataset, and 0.38% on the private dataset. Finally, the CBIS-DDSM dataset improved the shape classification results with 4.36% accuracy. Consequently, it is observed that the CAD system with the integrated detection and segmentation stages achieved much better results for the classification and diagnosis of breast masses.

**Table 13:** Comparison of classification performance (accuracy %) using the proposed CAD system with and without mass segmentation.

Dataset	Pathology		BI-RADS Category		Shape	
	CAD system without mass segmentation	CAD system with mass segmentation	CAD system without mass segmentation	CAD system with mass segmentation	CAD system without mass segmentation	CAD system with mass segmentation
CBIS-DDSM	90.87	95.13	81.69	85.38	85.66	90.02
INbreast	95.20	99.20	97.50	99	NA	NA
Private	90.38	95.88	95.70	96.08	NA	NA

Finally, a comparison of results of the latest state-of-the-art methods and similar models to classify the breast masses is listed in Table 14. Our proposed CAD system that integrates the previous detection and segmentation steps and the current proposed classification framework outperformed the previous deep learning models applied for pathology, BI-RADS category, and shape classification.

**Table 14:** Comparison of the proposed methodology and state-of-the-art methods.

Reference	Year	Method	Classification	Dataset	Images Segmented	Accuracy	AUC
^49^	2017	multi-view deep residual neural network (mResNet)	Pathology	INbreast	Yes	–	0.80
^51^	2018	Ensemble of AlexNet-based CNN	Pathology	INbreast	Yes	95.64	0.94
^25^	2019	MobileNet and NasNet + fine tuning	Pathology	CBIS-DDSM	Yes	78.4	–
^26^	2020	VGG16 + fine tuning	Pathology	CBIS-DDSM	No	84.4	0.84
^36^	2020	Improved Crow-Search Optimized Extreme Learning Machine (ICS-ELM) algorithm	Pathology	INbreast	No	98.26	–
^41^	2020	AlexNet, VGG, GoogLeNet, ResNet + fine tuning	Pathology	CBIS-DDSM	No	93.47	0.97
^43^	2020	DenseNet201 + MLP	BI-RADS	CBIS-DDSM	No	63.4	–
^42^	2020	NasNet + fine tuning on VGG16 and VGG19	BI-RADS	INbreast	No	90.9	0.99
^50^	2020	CNN	Shape	DDSM	Yes	80	0.80
^57^	2021	DualCoreNet: Texture and shape features fusion	Pathology	INbreast	Yes	–	0.93
Proposed CAD	2022	Stacked Ensemble of ResNet models	Pathology	CBIS-DDSM	Yes	95.13	0.95
				INbreast		99.20	0.99
				Private		95.88	0.95
			BI-RADS	CBIS-DDSM	Yes	85.38	0.94
				INbreast		99	1.00
				Private		96.08	0.95
			Shape	CBIS-DDSM	Yes	90.02	0.98

Compared with other techniques that used segmented ROIs, we exceeded the performance of the work by Falconí et al.^25^ that only achieving an accuracy of 78.4% using the MobileNet model on the CBIS-DDSM dataset. On the other hand, we also outperformed the work of Alkhaleefah et al.^41^ even though they did not use segmented input images and reported an accuracy of 93.47%. Moreover, recent works on the INbreast dataset were all surpassed where the highest accuracy of 98.26% was reported by Chakravarthy et al.^36^ using the ICS-ELM algorithm on original ROI masses. We also reported a better accuracy score for the pathology classification applied on segmented ROIs from the INbreast dataset, where Al-Antari et al.^51^ only achieved an accuracy of 95.64% and an AUC score of 0.94. The results of the BI-RADS categorization also outperformed the previous works on the CBIS-DDSM dataset with the work suggested by Medeiros et al.^43^ that applied DenseNet201 on original ROI masses and only achieved an accuracy of 63.4%. No previous paper applied the BI-RADS category classification to segmented images and therefore we could not compare it with our proposed work. Accordingly, our method surpassed the performance on the INbreast dataset by the work of Falconí et al.^42^ only reported an accuracy of 90.9% using NasNet and VGG models. Lastly, our methodology gained the best shape classification performance compared to a recent work of Singh et al.^50^ that applied a CNN model on a similar dataset, and it is reasonable to say that this reviewed work is the only comparable work that applied shape classification on detected and segmented ROIs but only achieved an accuracy of 80%.

## Discussion and conclusion

Deep learning models have recently revealed remarkable success in breast mass classification and diagnosis for many CAD systems. The CNN architecture model was mostly modified on many proposed studies and combined with other recent techniques such as transfer learning and ensemble model learning for better classification performance.

In this study, we have implemented a stacked ensemble of ResNet models to classify breast masses as malignant or benign and diagnose their BI-RADS category assessment with a score from 2 to 6 and their shape as oval, round, lobulated or irregular. The results of the proposed methodology showed the classification performance’s improvement compared to the individual architectures and the other methods applied to the existing benchmark datasets. Table 14 shows that we achieved the highest pathology classification performance on the two public datasets: CBIS-DDSM with an accuracy of 95.13% and an AUC score of 0.95, and INbreast with an accuracy of 99.20 and an AUC score of 0.99. Furthermore, we surpassed the results of other models for the BI-RADS categorization on the CBIS-DDSM dataset with an accuracy of 85.38% and an AUC score of 0.94, and on the INbreast dataset with an accuracy of 99% and an AUC score of 1.0. We also reported the highest results on the shape classification for the CBIS-DDSM dataset with an accuracy of 90.02% and an AUC score of 0.98.

Compared with the similar frameworks that applied the presented classification tasks on segmented ROI masses, our model outperformed the MobileNet and NasNet models^26^ for the pathology classification on the CBIS-DDSM dataset and the Ensemble of AlexNet-based CNN model^54^ on the INbreast dataset. Moreover, the shape classification achieved better results on a similar dataset DDSM that was evaluated with an individual CNN model^55^. As a result, the stacking model technique provided an efficient way to learn from various depths of neural networks and combine them in another neural network classifier model to benefit from the different weights that were trained individually.

The work integrated our recent works of the YOLO-based fusion models^28^ and the Connected-UNets model^53^ that generated the detected and segmented ROIs of breast masses. Indeed, an increase in performance using the segmented ROIs, as shown in Table 13, indicates the advantage of masking the background tissues from the tumors’ boundaries to help improve the overall classification and diagnosis, and decrease the false positive and negative rates. Limitations of the proposed methodology can occur on the long training time of 0.74 s per epoch, which is due to the high number of trainable parameters and computations of the ResNetV2 model.

In conclusion, this work presents the final stage of an integrated framework for a breast cancer CAD system via deep learning models. The three stages of detection, segmentation and classification aim to provide a complete clinical tool that can assist radiologists with a second suggestion for an automated mass tumor diagnosis. Future works can include combining different mammography datasets and improving the long training of deep learning models for the classification task.

## Supplementary Information

Supplementary Information.

## Data Availability

The public mammography dataset CBIS-DDSM generated and analyzed during the current study is available in the Cancer Imaging Archive, https://wiki.cancerimagingarchive.net/display/Public/CBIS-DDSM. The public mammography dataset INbreast generated and analyzed during the current study is available from the corresponding author Inês Domingues, Porto, Portugal, on reasonable request after signing a transfer agreement. The private mammography dataset generated during and analyzed during the current study is available from the corresponding author Cristian Castillo Olea through the oncologist Dr. Eric Ortiz in the National Institute of Cancerology, Mexico.

## Code availability

The code for custom algorithms and data preprocessing is provided as part of the replication package. It was written in Python v3.6. It is publicly available as a git repository on GitHub at https://github.com/AsmaBaccouche/Stacked-Ensemble-of-Residual-Neural-Networks.

## References

[1] Ferlay J (2021). Cancer statistics for the year 2020: An overview. Int. J. Cancer.

[2] American Cancer Society. Cancer Statistics Center. http://cancerstatisticscenter.cancer.org. Accessed January 17, 2022.

[3] Duffy SW (2020). Mammography screening reduces rates of advanced and fatal breast cancers: Results in 549,091 women. Cancer.

[4] Dibden A, Offman J, Duffy SW, Gabe R (2020). Worldwide review and meta-analysis of cohort studies measuring the effect of mammography screening programmes on incidence-based breast cancer mortality. Cancers.

[5] Rahman MM (2021). Machine learning based computer aided diagnosis of breast cancer utilizing anthropometric and clinical features. Irbm.

[6] Ramadan SZ (2020). Methods used in computer-aided diagnosis for breast cancer detection using mammograms: A review. J. Healthc. Eng..

[7] Yassin NI, Omran S, El Houby EM, Allam H (2018). Machine learning techniques for breast cancer computer aided diagnosis using different image modalities: A systematic review. Comput. Methods Programs Biomed..

[8] Paramkusham, S., Thotempuddi, J., & Rayudu, M. S. Breast masses classification using contour shape descriptors based on Beam Angle Statistics. In *2021 Third International Conference on Inventive Research in Computing Applications (ICIRCA)*, 1340–1345. (IEEE, 2021).

[9] Li H, Meng X, Wang T, Tang Y, Yin Y (2017). Breast masses in mammography classification with local contour features. Biomed. Eng. Online.

[10] Yuvaraj N (2021). Analysis of protein-ligand interactions of SARS-Cov-2 against selective drug using deep neural networks. Big Data Mining Anal..

[11] Hartpence B, Kwasinski A (2021). CNN and MLP neural network ensembles for packet classification and adversary defense. Intell. Converg. Netw..

[12] Al-Masni MA (2018). Simultaneous detection and classification of breast masses in digital mammograms via a deep learning YOLO-based CAD system. Comput. Methods Programs Biomed..

[13] Eltrass AS, Salama MS (2020). Fully automated scheme for computer-aided detection and breast cancer diagnosis using digitised mammograms. IET Image Proc..

[14] Siddiqui SY (2021). Intelligent breast cancer prediction empowered with fusion and deep learning. Computer Mater. Continua.

[15] Ragab M, Albukhari A, Alyami J, Mansour RF (2022). Ensemble deep-learning-enabled clinical decision support system for breast cancer diagnosis and classification on ultrasound images. Biology.

[16] Aly GH, Marey M, El-Sayed SA, Tolba MF (2021). YOLO based breast masses detection and classification in full-field digital mammograms. Comput. Methods Programs Biomed..

[17] Al-Antari MA, Han SM, Kim TS (2020). Evaluation of deep learning detection and classification towards computer-aided diagnosis of breast lesions in digital X-ray mammograms. Comput. Methods Programs Biomed..

[18] Abdelhafiz D, Bi J, Ammar R, Yang C, Nabavi S (2020). Convolutional neural network for automated mass segmentation in mammography. BMC Bioinform..

[19] Taghanaki SA (2021). Deep semantic segmentation of natural and medical images: A review. Artif. Intell. Rev..

[20] Jiao Z, Gao X, Wang Y, Li J (2018). A parasitic metric learning net for breast mass classification based on mammography. Pattern Recogn..

[21] Xu X, Gao T, Wang Y, Xuan X (2021). Event temporal relation extraction with attention mechanism and graph neural network. Tsinghua Sci. Technol..

[22] Gu W, Gao F, Li R, Zhang J (2021). Learning universal network representation via link prediction by graph convolutional neural network. J. Soc. Comput..

[23] Rasti R, Teshnehlab M, Phung SL (2017). Breast cancer diagnosis in DCE-MRI using mixture ensemble of convolutional neural networks. Pattern Recogn..

[24] Rampun, A., Scotney, B. W., Morrow, P. J., & Wang, H. Breast mass classification in mammograms using ensemble convolutional neural networks. In *2018 IEEE 20th International Conference on e-Health Networking, Applications and Services (Healthcom)*, 1–6 (IEEE 2018).

[25] Falconí, L. G., Pérez, M., & Aguilar, W. G. Transfer learning in breast mammogram abnormalities classification with mobilenet and nasnet. In *2019 International Conference on Systems, Signals and Image Processing (IWSSIP)*, 109–114 (IEEE, 2019).

[26] Falconi LG, Perez M, Aguilar WG, Conci A (2020). Transfer learning and fine tuning in breast mammogram abnormalities classification on CBIS-DDSM database. Adv. Sci. Technol. Eng. Syst..

[27] Ragab DA, Attallah O, Sharkas M, Ren J, Marshall S (2021). A framework for breast cancer classification using multi-DCNNs. Comput. Biol. Med..

[28] Baccouche A, Garcia-Zapirain B, Castillo Olea C, Elmaghraby AS (2021). Breast lesions detection and classification via yolo-based fusion models. Comput. Mater. Contina.

[29] Zahoor S, Shoaib U, Lali IU (2022). Breast cancer mammograms classification using deep neural network and entropy-controlled Whale optimization algorithm. Diagnostics.

[30] Dhahri H, Rahmany I, Mahmood A, Al Maghayreh E, Elkilani W (2020). Tabu search and machine-learning classification of benign and malignant proliferative breast lesions. BioMed Res. Int..

[31] Shen L (2019). Deep learning to improve breast cancer detection on screening mammography. Sci. Rep..

[32] Shams, S. *et al*. Deep generative breast cancer screening and diagnosis. In *International Conference on Medical Image Computing and Computer-Assisted Intervention*, 859–867 (Springer, Cham 2018).

[33] Li H, Zhuang S, Li DA, Zhao J, Ma Y (2019). Benign and malignant classification of mammogram images based on deep learning. Biomed. Signal Process. Control.

[34] Zhang Q (2020). A novel algorithm for breast mass classification in digital mammography based on feature fusion. J. Healthc. Eng..

[35] Muramatsu C (2020). Improving breast mass classification by shared data with domain transformation using a generative adversarial network. Comput. Biol. Med..

[36] Chakravarthy SS, Rajaguru H (2022). Automatic detection and classification of mammograms using improved extreme learning machine with deep learning. IRBM.

[37] Khan HN, Shahid AR, Raza B, Dar AH, Alquhayz H (2019). Multi-view feature fusion based four views model for mammogram classification using convolutional neural network. IEEE Access.

[38] Jasti V (2022). Computational technique based on machine learning and image processing for medical image analysis of breast cancer diagnosis. Secur. Commun. Netw..

[39] Kumar I, Bhadauria HS, Virmani J, Thakur S (2017). A classification framework for prediction of breast density using an ensemble of neural network classifiers. Biocybern. Biomed. Eng..

[40] Yurttakal, A. H., Erbay, H., İkizceli, T., Karaçavuş, S., & Biçer, C. Diagnosing breast cancer tumors using stacked ensemble model. *J. Intell. Fuzzy Syst*. Preprint at https://content.iospress.com/articles/journal-of-intelligent-and-fuzzy-systems/ifs219176 (2022).

[41] Alkhaleefah M (2020). Double-shot transfer learning for breast cancer classification from X-ray images. Appl. Sci..

[42] Falconí, L., Pérez, M., Aguilar, W., & Conci, A. Transfer Learning and Fine Tuning in Mammogram BI-RADS Classification. In *2020 IEEE 33rd International Symposium on Computer-Based Medical Systems (CBMS)*, 475–480 (IEEE, 2020).

[43] Medeiros, A., Ohata, E. F., Silva, F. H., Rego, P. A., & Reboucas Filho, P. P. An approach to BI-RADS uncertainty levels classification via deep learning with transfer learning technique. In *2020 IEEE 33rd International Symposium on Computer-Based Medical Systems (CBMS)*, 603–608 (IEEE, 2020).

[44] Tsai KJ (2022). A high-performance deep neural network model for BI-RADS classification of screening mammography. Sensors.

[45] Bi WL (2019). Artificial intelligence in cancer imaging: Clinical challenges and applications. CA A Cancer J. Clin..

[46] Tsochatzidis L, Koutla P, Costaridou L, Pratikakis I (2021). Integrating segmentation information into CNN for breast cancer diagnosis of mammographic masses. Comput. Methods Programs Biomed..

[47] Li, H., Chen, D., Nailon, W. H., Davies, M. E., & Laurenson, D. Dual Convolutional Neural Networks for Breast Mass Segmentation and Diagnosis in Mammography. Preprint at https://arxiv.org/abs/2008.02957 (2020). 10.1109/TMI.2021.3102622 34351855

[48] Sarkar, P. R., Prabhakar, P., Mishra, D., & Subrahmanyam, G. Towards automated breast mass classification using deep learning framework. In *2019 IEEE International Conference on Data Science and Advanced Analytics (DSAA)*, 453–462 (IEEE, 2019).

[49] Dhungel, N., Carneiro, G., & Bradley, A. P. Fully automated classification of mammograms using deep residual neural networks. In *2017 IEEE 14th International Symposium on Biomedical Imaging (ISBI 2017)*, 310–314 (IEEE, 2017).

[50] Singh VK (2020). Breast tumor segmentation and shape classification in mammograms using generative adversarial and convolutional neural network. Expert Syst. Appl..

[51] Al-Antari MA, Al-Masni MA, Choi MT, Han SM, Kim TS (2018). A fully integrated computer-aided diagnosis system for digital X-ray mammograms via deep learning detection, segmentation, and classification. Int. J. Med. Informatics.

[52] Al-Antari MA, Al-Masni MA, Kim TS (2020). Deep learning computer-aided diagnosis for breast lesion in digital mammogram. Deep Learn. Med. Image Anal..

[53] Baccouche A, Garcia-Zapirain B, Castillo Olea C, Elmaghraby AS (2021). Connected-UNets: A deep learning architecture for breast mass segmentation. NPJ Breast Cancer.

[54] He, K., Zhang, X., Ren, S., & Sun, J. Deep residual learning for image recognition. In *Proceedings of the IEEE Conference on Computer Vision and Pattern Recognition*, 770–778 (2016)

[55] Garcia-Gasulla D (2018). On the behavior of convolutional nets for feature extraction. J. Artif. Intell. Res..

[56] Szegedy, C., Ioffe, S., Vanhoucke, V., & Alemi, A. A. Inception-v4, inception-resnet and the impact of residual connections on learning. In *Thirty-first AAAI Conference on Artificial Intelligence* (2017).

[57] Yu X (2020). ResNet-SCDA-50 for breast abnormality classification. IEEE/ACM Trans. Comput. Biol. Bioinform..

[58] He, K., Zhang, X., Ren, S., & Sun, J. Identity mappings in deep residual networks. In *European Conference on Computer Vision*, 630–645 (Springer, Cham 2016).

[59] Chen, Y. et al. Fine-tuning ResNet for breast cancer classification from mammography. In *The International Conference on Healthcare Science and Engineering*, 83–96 (Springer, Singapore 2018).

[60] Bellmann, P., Thiam, P., & Schwenker, F. Multi-classifier-systems: architectures, algorithms and applications. In *Computational Intelligence for Pattern Recognition*, 83–113 (Springer, Cham 2018).

[61] Lee RS (2017). A curated mammography data set for use in computer-aided detection and diagnosis research. Sci. Data.

[62] Moreira IC (2012). Inbreast: toward a full-field digital mammographic database. Acad. Radiol..

[63] Müller, R., Kornblith, S., & Hinton, G. When does label smoothing help?. Preprint at https://arxiv.org/abs/1906.02629 (2019).

