# Supplementary material for: An integrated framework for breast mass classification and diagnosis using stacked ensemble of residual neural networks
Source: Sci Rep. 2022 Jul 18;12:12259. doi: 10.1038/s41598-022-15632-6 (PMC9293883; doi:10.1038/s41598-022-15632-6)
Supplement: Supplementary file 1 — Supplementary Information. [file 41598_2022_15632_MOESM1_ESM.pdf]

An integrated Framework for Breast Mass Classification and Diagnosis using Stacked Ensemble of Residual Neural Networks

Asma Baccouche[1], Begonya Garcia-Zapirain[2] and Adel S. Elmaghraby[1]

[1]Department of Computer Science and Engineering, University of Louisville, Louisville, KY, USA, 40292

[2]eVida Research Group, University of Deusto, Bilbao, Spain, 4800

Email address for corresponding author: asma.baccouche@louisville.edu

Supplementary Methods

As shown in Supplementary Figure 1, the improved version of ResNet architecture presented as ResNetV2 by He et al. where the last ReLU was removed to clear the shortcut path using a simple identity connection. All ResNet architectures use the same residual blocks containing three convolutional layers with stride 1 and 3 followed by a BatchNormalization (BN) layer and a non-linearity layer Rectified Linear Unit (ReLU) on its shortcut path.

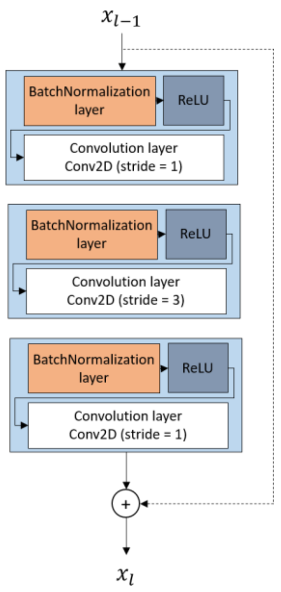

Supplementary Figure.1: Residual block of ResNetV2

The architectures accept RGB input images of size 224 x 224 and they are composed of an initial convolutional layer with a filter size 7 x 7 and stride 2, followed by a pooling layer of size 3 x 3 and stride

2. The base architecture should then use four residual blocks with different kernel sizes of 64, 128, 256, and 512. Each architecture has a different number of residual blocks that define the depth of layers. Finally, a global average pooling layer with size 7 x 7 is used with a final fully connected layer (FC).

Supplementary Table.1: ResNetV2 architecture layers

Layer Name	Output Size	ResNet50V2	ResNet101V2	ResNet152V2
Input Layer		224 x 224 x 3		
conv1	112 x 112 x 64	Number of filters = 64<br>Filter size = 7 x 7<br>Strides = 2<br>Padding = 3		
pool1	56 x 56 x 64	Strides = 2<br>Pooling size = 3 x 3		
conv2_x	56 x 56 x 64	$\begin{bmatrix} 1 \times 1, & 64 \\ 3 \times 3, & 64 \\ 1 \times 1, & 256 \end{bmatrix}$ x 3	$\begin{bmatrix} 1 \times 1, & 64 \\ 3 \times 3, & 64 \\ 1 \times 1, & 256 \end{bmatrix}$ x 3	$\begin{bmatrix} 1 \times 1, & 64 \\ 3 \times 3, & 64 \\ 1 \times 1, & 256 \end{bmatrix}$ x 3
conv3_x	28 x 28 x 128	$\begin{bmatrix} 1 \times 1, & 128 \\ 3 \times 3, & 128 \\ 1 \times 1, & 512 \end{bmatrix}$ x 4	$\begin{bmatrix} 1 \times 1, & 128 \\ 3 \times 3, & 128 \\ 1 \times 1, & 512 \end{bmatrix}$ x 4	$\begin{bmatrix} 1 \times 1, & 128 \\ 3 \times 3, & 128 \\ 1 \times 1, & 512 \end{bmatrix}$ x 8
conv4_x	14 x 14 x 256	$\begin{bmatrix} 1 \times 1, & 256 \\ 3 \times 3, & 256 \\ 1 \times 1, & 1024 \end{bmatrix}$ x 6	$\begin{bmatrix} 1 \times 1, & 256 \\ 3 \times 3, & 256 \\ 1 \times 1, & 1024 \end{bmatrix}$ x 23	$\begin{bmatrix} 1 \times 1, & 256 \\ 3 \times 3, & 256 \\ 1 \times 1, & 1024 \end{bmatrix}$ x 36
conv5_x	7 x 7 x 512	$\begin{bmatrix} 1 \times 1, & 512 \\ 3 \times 3, & 512 \\ 1 \times 1, & 2048 \end{bmatrix}$ x 3	$\begin{bmatrix} 1 \times 1, & 512 \\ 3 \times 3, & 512 \\ 1 \times 1, & 2048 \end{bmatrix}$ x 3	$\begin{bmatrix} 1 \times 1, & 512 \\ 3 \times 3, & 512 \\ 1 \times 1, & 2048 \end{bmatrix}$ x 3
Global average pooling	2048	Pooling size = 7 x 7<br>Strides = 7		
Fully connected	1000	1 x 1 x 2048		
